# Fabrication of Multi-Layered Microspheres Based on Phase Separation for Drug Delivery

**DOI:** 10.3390/mi12060723

**Published:** 2021-06-19

**Authors:** He Xia, Ang Li, Jia Man, Jianyong Li, Jianfeng Li

**Affiliations:** 1 Key Laboratory of High Efficiency and Clean Mechanical Manufacture of MOE, School of Mechanical Engineering, Shandong University, Jinan 250061, China; xiahe@mail.sdu.edu.cn (H.X.); ljy@sdu.edu.cn (J.L.); ljf@sdu.edu.cn (J.L.); 2 Key National Demonstration Center for Experimental Mechanical Engineering Education, Shandong University, Jinan 250061, China; 3 School of Intelligent Engineering, Shandong Management University, Changqing, Jinan 250357, China; la@sdmu.edu.cn

**Keywords:** PEGDA, microfluidic, phase separation, microsphere, drug delivery

## Abstract

In this work, we used a co-flow microfluidic device with an injection and a collection tube to generate droplets with different layers due to phase separation. The phase separation system consisted of poly(ethylene glycol) diacrylate 700 (PEGDA 700), PEGDA 250, and sodium alginate aqueous solution. When the mixture droplets formed in the outer phase, PEGDA 700 in the droplets would transfer into the outer aqueous solution, while PEGDA 250 still stayed in the initial droplet, breaking the miscibility equilibrium of the mixture and triggering the phase separation. As the phase separation proceeded, new cores emerged in the droplets, gradually forming the second and third layers. Emulsion droplets with different layers were polymerized under ultraviolet (UV) irradiation at different stages of phase separation to obtain microspheres. Microspheres with different layers showed various release behaviors in simulated gastric fluid (SGF) and simulated intestinal fluid (SIF). The release rate decreased with the increase in the number of layers, which showed a potential application in sustained drug release.

## 1. Introduction

Microparticles with complex structures, such as core-shell [1,2], porous [3], Janus [4,5], and multi-layered structures [6], have promising applications in drug delivery [7,8,9], cell encapsulation [8,10,11], cosmetics [12,13], and biosensors [14]. Droplet microfluidics provides a unique method for the fabrication of monodisperse microparticles with control over the size, morphology, and functionality in a high throughput manner [15]. Droplets can be converted into solid microparticles by polymerization [16], ionic crosslinking [17,18], solvent evaporation [16], etc. Among the applications of microparticles, the encapsulation of drugs with biocompatible polymers has attracted the interest of many researchers in recent years. The encapsulation of the drug in polymeric microparticles or microfibers allows a sustained and slow release of drugs, preventing premature metabolism of the drug in the organism [19]. Many researchers have studied the application of microparticles with different shapes in drug delivery. It has been shown that many drugs need to be administered at varying rates, and for some drugs, such as those used at the beginning of wound treatment, an initial burst provides immediate relief followed by prolonged release to promote gradual healing [20]. Huang et al. studied silk fibroin/alginate microspheres for rapid hemostasis. Both in vitro and in vivo coagulation experiments demonstrated that the burst release of the drug in microspheres could reduce bleeding time and volume and consequently improve hemostatic efficiency [21]. For some drugs, such as those used for long-term treatment, sustained release was needed to prolong the time that the drug remains in the body at the therapeutic level. Hayashi et al. prepared red blood cell-shaped microparticles with a red blood cell membrane and demonstrated prolonged circulation time in blood [22]. Zhang et al. studied the preparation and evaluation of alginate–chitosan microspheres for oral delivery of insulin, hoping to maintain proper glucose concentration for a long time, which would bring convenience to diabetics [23]. 

**Figure 1:**
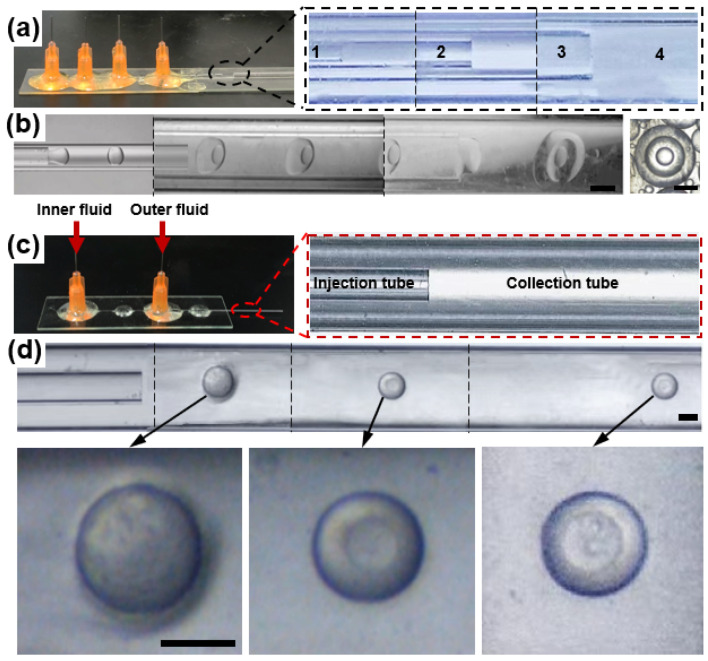
(**a**) Digital photographs of the microfluidic device with four cylindrical glass capillary tubes. From the inside out, each number represents a glass capillary. To make droplets with three layers, four cylindrical glass capillary tubes are needed to be arrayed into a critical structure. (**b**) The optical microscopic image of the process to produce droplets with three layers. The droplet produced by the device is shown to the right. Scale bar = 500 µm. (**c**) Microfluidic device with a co-flow structure. It was composed of two cylindrical glass capillary tubes called the injection tube and the collection tube, respectively. When using it to prepare droplets, inner fluid and outer fluid are injected into the injection tube and the collection tube by syringe pumps, respectively. (**d**) The optical microscopic image of the process to produce droplets with different layers. Scale bar = 200 µm.

A distinctive feature of microfluidics is that the combination of single-stage channels can be upgraded to a complex microchannel network system, providing conditions for the controlled construction of double and multiple emulsion droplets [24]. Single emulsion droplets are usually produced by two immiscible liquid phases, with one phase dispersed into the second [25], the double emulsion can be viewed as the dispersion of a primary emulsion in another aqueous or oil phase [26,27,28], and multiple emulsion can be formed by multiple-step emulsification of multiphase fluids in microchannels [29]. In order to prepare multi-layered microparticles, a microfluidic device with several nested microchannels is usually required. For instance, to make droplets with three layers, four cylindrical glass capillary tubes are needed, as shown in Figure 1a. The flow rate of each phase needs to be precisely controlled to ensure that the droplets in the previous channel can be successfully encapsulated, as shown in Figure 1b. In addition, precise and complicated hydrophilic/hydrophobic treatment of the microchannels is also required according to the property of multiple droplets [24]. 

In recent years, liquid−liquid phase separation has been used to prepare multi-layered micro-emulsion droplets [6,30,31]. This technology refers to the phenomenon that when the external environment changes, the miscibility of several solutions dissolved together changes, and a certain phase or several phase solutions separate from the mixed solution [6]. Usually, a homogeneous emulsion droplet can subsequently generate complex emulsions due to phase separation [32]. At present, the external factors that induce the phase separation are mainly mass transfer between the droplet and external solution [33], temperature [34], and polymerization [35], etc. Once the experimental conditions such as liquid ratio and temperature are set, phase separation can proceed spontaneously, so phase separation is also considered an intelligent process. Compared with using the complex microfluidic device to control the structure of microparticles, phase separation technology is easy to operate. By mixing hexane with perfluorohexane, for instance, Zarzar et al. succeeded in transforming single emulsion droplets into multi-layered and Janus-like droplets by changing the temperature [34]. Guo et al. found that the polymerization of polymers could also induce phase separation. They dissolved polyethylene glycol and acrylamide in water and found that with the increase in temperature, acrylamide polymerized, and a core of polyacrylamide was gradually formed inside the droplet, thus separating into a core-shell structure of particles [36]. Some polymers are often used in phase separation systems, such as poly(methyl methacrylate) (PMMA) [37] and some light-curable materials like poly(ethylene glycol) diacrylate (PEGDA) and ethoxylated trimethylolpropane triacrylate (ETPTA) [38,39]. When preparing microparticles using microfluidic devices, the size of microparticles can be controlled by adjusting the flow rate of each phase [39,40], and the shape of the microparticles can be controlled by adjusting the ingredient content, surfactant content, and surfactant type to change interfacial tension [38]. Hence, phase separation technology can realize the preparation of complex microparticles by using a microfluidic device with a simple structure, and microparticles with different structures can be obtained by changing the parameters. However, the material system of phase separation still needs to be explored and studied, and, in addition, few studies have been conducted on the properties of the drug delivery of microparticles with different layers after phase separation.

Herein, we combined a co-flow microfluidic device (Figure 1c) with phase separation to firstly generate single emulsion droplets, and then we prepared microspheres with different layers for drug delivery, as shown in Figure 1d. We selected PEGDA, whose properties such as hydrophilicity, crosslinking density, and mechanical strength are determined by molecular weight [41], as the ingredients of the phase separation system. PEGDA can be immediately cured by UV and it is non-toxic and biodegradable [42], and it has been approved for clinical use by the U.S. Food and Drug Administration (FDA). The smaller the molecular weight of PEGDA, the more hydrophobic it is [43], resulting in PEGDA 250 being hydrophobic, while PEGDA 700 is soluble in both oil and water. Calcium alginate hydrogel prepared from sodium alginate, a plant extract of brown algae, has good biocompatibility and biodegradability, making it have great potential application in drug carriers [44] and surgical dressings [45]. Thus, we used PEGDA 700 as a co-solvent in the phase separation system, and PEGDA 250 and sodium alginate aqueous solution as two immiscible phases. The microspheres were fabricated from a microfluidic device with an easy co-flow structure by using the mixed solution consisting of these three contents as the inner phase and poly (vinyl alcohol) (PVA) aqueous solution as the outer phase. As PEGDA 700 is miscible with both PEGDA 250 and water, part of PEGDA 700 was rapidly transferred from the initial droplet to the external aqueous solution once the single droplet was prepared in PVA aqueous solution. Therefore, mass transfer-induced phase separation took place, forming droplets with one, two, and three layers. Droplets could be converted into solid microspheres with different layers by UV curing at different times during the phase separation process. Furthermore, the model drug was loaded in the microspheres and it was found that microspheres with different layers have different release rates, which can provide a method in designing particles with a controlled drug release profile for drug delivery.

## 2. Materials and Methods

### 2.1. Materials

The mixture of PEGDA 700 (M_n_ = 700, SigmaAldrich, St. Louis, MO, USA), PEGDA 250 (Mn = 700, Sigma Aldrich, St. Louis, MO, USA), and sodium alginate (Macklin Chemical Reagent Shanghai Co., Ltd, Shanghai, China) (1 wt%) aqueous solution (Na-Alginate) was used as the inner phase fluid. 2-Hydroxy-2-methylpropiophenone (2% *v*/*v*) was added into the mixture of PEDGA 700 and PEDGA 250 as the photoinitiator for UV light curing. Poly (vinyl alcohol) (PVA) (97.5–99% hydrolyzed, Aladdin Reagent Shanghai Co., Ltd, Shanghai, China) was added into deionized water (DI) to achieve a concentration of 5 wt% to be used as the continuous phase fluid. Doxorubicin hydrochloride (DOX) was used as a model drug. Simulated gastric fluid (SGF, pH = 1.2, Shanghai Yuanye Bio-Technology Co., Ltd. Shanghai, China) and simulated intestinal fluid (SIF, pH = 7.2, Shanghai Yuanye Bio-Technology Co., Ltd. Shanghai, China) were used to simulate the environment of the human digestive tract in the drug release experiment. Silicone oil (Aladdin Reagent Shanghai Co., Ltd, Shanghai, China; 20 mPa.s) was used as the oil phase to obtain homogeneous “cup-shaped” microparticles.

### 2.2. Microfluidic Device Setup

The microfluidic device had a co-flow structure, which was composed of two cylindrical glass capillary tubes called an injection tube and collection tube, respectively. The injection glass tube with an inner diameter of 300 µm was coaxially positioned inside the collection tube with an inner diameter of 900 µm. Two glass tubes were fixed on the glass sheet with epoxy glue, and the entrance of each glass tube was connected with a dispensing needle, as shown in Figure 1c. 

### 2.3. Preparation of Emulsion Droplets with Different Layers

The inner and continuous phase fluids were injected into the injection tube and the collection tube, respectively. Each liquid was connected with a syringe pump (Longer Pump LSP01-3A) through a polyethylene tube (Scientific Commodities Inc, Lake Havasu City, AZ, USA). The initial single emulsion can be obtained due to the shear force of the outer fluid to the inner fluid. As the droplets flowed downstream, PEGDA 700 in the droplets would transfer into the outer aqueous solution, while PEGDA 250 still stayed in the initial droplet, breaking the miscibility equilibrium of the mixture and triggering the phase separation. As the phase separation proceeded, new cores emerged in the droplets, gradually forming the second and third layers. Different periods of the phase separation process were captured using the microscope (Leica DM4B, Leica Camera, Wetzlar, Germany). The inner mixture was dyed with Nile red in order to provide distinct images for the investigation of the mass transfer process during phase separation.

### 2.4. Preparation of Microparticles with Different Layers

For the preparation of microparticles with different layers, a 2 *v*/*v*% photoinitiator was added into the inner fluid, and an ultraviolet beam was applied over the downstream of the PTFE tube. PEGDA 700 and PEGDA 250 are both photocurable, so the emulsion droplets were polymerized under UV irradiation at different stages of phase separation to obtain microparticles with different layers. The cured microparticles were washed by DI water and were dried at room temperature for at least 12 h.

### 2.5. Drug Loading and Controlled Release

The drug of DOX hydrochloride (500 μg mL^–1^) was dissolved in the inner mixture. For controlled release, the DOX-loaded microparticles were dispersed in a cuvette of 4 mL simulated gastric fluid (SGF) for 2 h and 4 mL simulated intestinal fluid (SIF) for six hours at room temperature. The concentration of DOX was measured using an ultraviolet−visible spectrophotometer at a wavelength of maximum absorbance (480 nm) at regular intervals.

### 2.6. Characterization

Leica DFC450 C camera with a green fluorescence module (Leica 11513878 BZ: 01) was used to image all experiments which were performed on a microscope (Leica DM4B). The morphology of dried microparticles was observed by scanning electron microscope (SEM, JSM-6610LV) using an accelerating voltage of 5 kV, and the samples were coated with gold before measurement. A digital microscopic system (Keyence VHX-2000) was also used to measure the size of the microparticles. Varian Cary 100 Bio UV−visible spectrophotometer was used to measure the concentration of DOX.

## 3. Results and Discussion

### 3.1. Preparation of Emulsion Droplets with Different Layers

**Figure 2:**
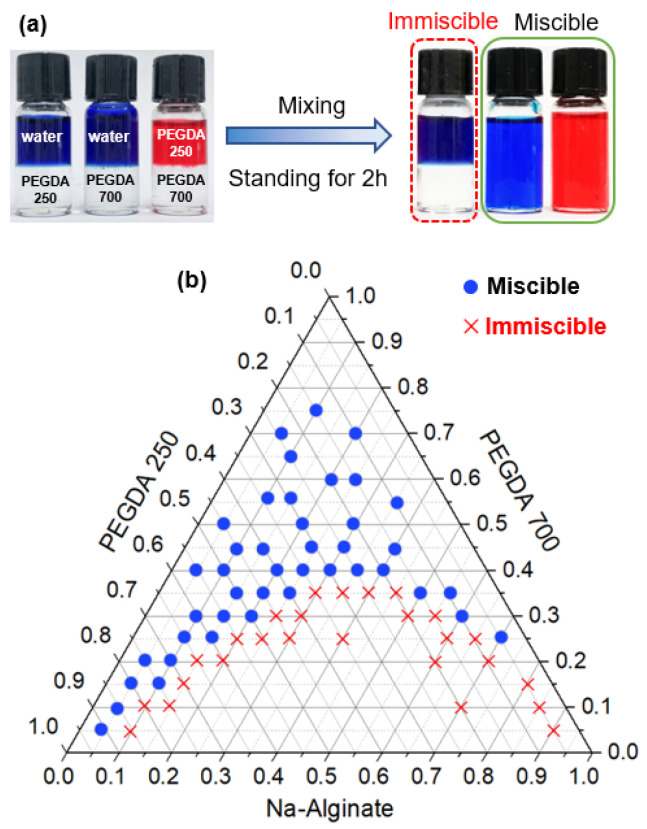
(**a**) Digital photographs showing the miscibility of three liquids. The deionized water (DI) water contained a water-soluble blue dye, and poly(ethylene glycol) diacrylate 250 (PEGDA 250) contained an oil-soluble red dye when mixed with PEGDA 700. After enough time for mixing, bottles stood for 2 h. PEGDA 250 was immiscible with water, whereas PEGDA 700 was miscible with both PEGDA 250 and water. (**b**) Ternary phase diagram illustrating the solubility of three solutions in this phase separation system. The blue dots mean that the three solutions were miscible at that volume ratio and the red crosses indicate that the three solutions were immiscible at that volume ratio.

Mass transfer-induced liquid–liquid phase separation requires at least two immiscible liquids and one co-solvent. PEGDA contains double-bond acrylate groups at each end of the PEG chain [46] and is a light-curable material. PEGDA is available in various MWs, making it ideal for obtaining the required hydrophilicity or hydrophobicity, and the smaller the molecular weight of PEGDA, the more hydrophobic the polymer [43]. Through the experiments, we found that PEGDA 250 was immiscible with water, but PEGDA 700 was miscible with both PEGDA 250 and water. To make the phenomenon easier to observe, we mixed PEGDA 250 with water, PEGDA 700 with water, and PEGDA 250 with PEGDA 700, respectively, in small glass bottles, as shown in Figure 2a. The DI water contained a water-soluble blue dye, and PEGDA 250 contained an oil-soluble red dye when mixed with PEGDA 700. After enough time for mixing, the bottles stood for 2 h, and we observed that PEGDA 250 and water formed two distinctive layers, while the liquids in the other two bottles were miscible. In order to know the miscibility of PEGDA 700, PEGDA 250, and Na-Alginate, we mixed three liquids in different proportions in a centrifuge tube to a total volume of 1 mL. The miscibility of this phase separation system is represented in a ternary phase diagram, which can be used to analyze the process of droplet phase separation, as shown in Figure 2b. The blue dots mean that the three solutions were miscible at that volume ratio and the red crosses indicate that the three solutions were immiscible at that volume ratio.

**Figure 3:**
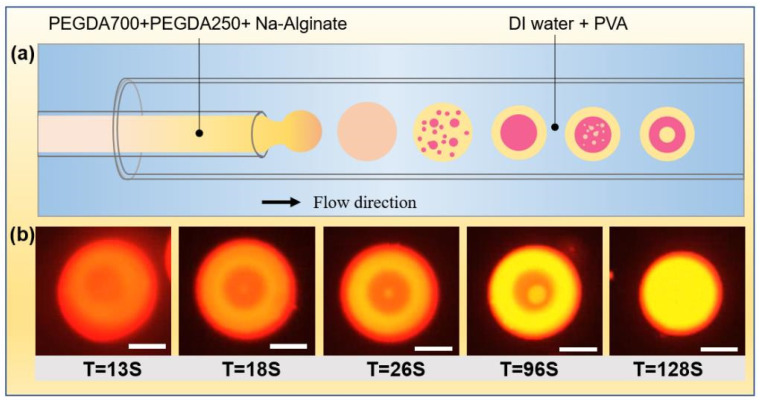
(**a**) Schematics of the microfluidic device used for preparing initial single emulsion droplet and the droplet changed from one layer to two and three layers. (**b**) Formation of single, double, and triple emulsion droplets over time due to phase separation. The scale bar is 100 µm. The hydrophobic fluorescent indicator Nile red was premixed into the inner phase mixture and automatically assembled into the PEGDA 250-rich layer during the phase separation process. The PEGDA 250-rich layer was labeled with a bright yellow fluorescent region.

A microfluidic device with a co-flow structure was used to prepare single emulsion droplets of different sizes, as shown in Figure 3a. We used a mixture containing 50 *v*/*v*% PEGDA 250, 40 *v*/*v*% PEGDA 700, and 10 *v*/*v*% Na-Alginate aqueous solution as the inner phase and 5 wt% PVA aqueous solution as the continuous phase. Additionally, the flow rates of the inner and outer fluid were 0.5 mL/h and 5 mL/h, respectively. Once the droplets formed at the outlet of the injection tube, PEGDA 700 in the droplet gradually diffused to the external solution. Therefore, the miscibility equilibrium between PEGDA 250 and the aqueous solution was broken, leading to the phase separation. We recorded the droplet phase separation process by microscopy, as shown in Figure 3b. The hydrophobic fluorescent indicator Nile red was premixed into the inner phase mixture and automatically assembled into the PEGDA 250-rich layer during the phase separation process. The PEGDA 250-rich layer was labeled with a bright yellow fluorescent region, making it clear to recognize the distribution of different compositions in droplets. As the phase separation proceeded, the original single emulsion droplet gradually separated into a bilayered emulsion droplet and progressively formed a tri-layer emulsion droplet. During the phase separation, the PEGDA 250-rich phase and the PEGDA 700-rich phase were alternated.

**Figure 4:**
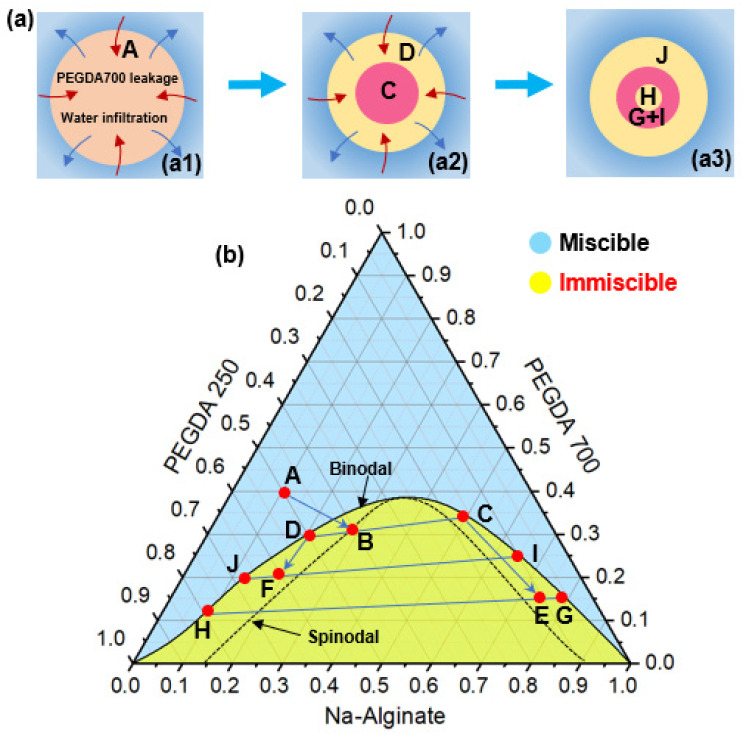
(**a**) Schematic of the change process of emulsion droplets in phase separation: single (**a1**), double (**a2**) and triple (**a3**) emulsion. (**b**) Ternary phase diagram for analyzing the phase separation process. The blue region means that the three solutions were miscible at that volume ratio, and the yellow region indicates that the three solutions were immiscible at that volume ratio.

The phase separation process of droplets can be analyzed by using the phase diagrams of PEGDA 250, PEGDA 700, and Na-Alginate solution. As shown in Figure 4b, the blue region means that the three solutions were miscible at that volume ratio, and the yellow region indicates that the three solutions were immiscible at that volume ratio. According to the solubility of three liquids, the binodal line in the phase diagram could be determined through experiments to represent the boundary between the miscible and immiscible states. The spinodal line in the phase diagram was an imaginary curve to represent the spontaneous phase separation in this state. The droplet was made from a mixture of PEGDA250, PEGDA 700, and Na-Alginate solution with a volume ratio of 5:4:1, and it was located at point A in the diagram. When the droplet entered the external aqueous solution, PEGDA 700 decreased, and water infiltrated (Figure 4(a1)). The volume ratio gradually moved from point A across the binodal line to point B on the spinodal line, causing phase separation in the droplet to begin. The droplet would spontaneously form a PEGDA 700-rich phase and a PEGDA 250-rich phase, whose volume ratios were located at points C and D of the phase diagram, respectively, resulting in a double emulsion shown in Figure 4(a2). According to the level rule in phase separation [47], both points C and D were located on the binodal line of the phase diagram. As PEGDA 700 continued to decrease, the composition of point C continued to move to point E and then spontaneously separated into a PEGDA 250-rich phase H and a PEGDA 700-rich phase G. Meanwhile, point D would continue to move to point F and then spontaneously separated a PEGDA 700-rich phase I and aqueous solution and a PEGDA 250-rich phase J. At this moment, the state of the droplet is a triple emulsion droplet, as shown in Figure 4(a3).

### 3.2. Preparation of Microparticles with Different Layers

**Figure 5:**
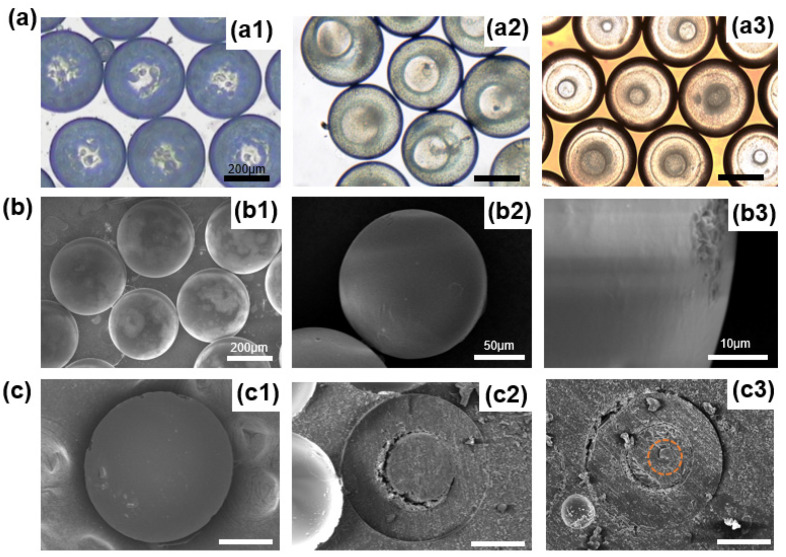
(**a**) Optical microscopic images of microspheres with different numbers of layers: (**a1**) One layer, (**a2**) Two layers, (**a3**) Three layers. The scale bar is 200 µm. (**b**) Scanning electron microscope images of microspheres prepared at one flow rate ratio. (**b1**) SEM image showing good monodispersity and sphericity of microspheres; (**b2**) Magnified SEM image of the microsphere; (**b3**) SEM image showing the solid, smooth, and dense surface of the microsphere. (**c**) Scanning electron microscope images of different number-layered microspheres cut open: (**c1**) One layer, (**c2**) Two layers, (**c3**) Three layers. The scale bar is 100 µm.

Depending on the droplet structure at different times, microspheres with one, two, or three layers could be obtained by adjusting the position of UV light irradiation. We collected microspheres in a Petri dish with different layers by curing them at 10 s, 30 s, and 120 s, respectively, and observed them in a microscope to record the structure and size of microspheres, as shown in Figure 5(a1–a3). It can be seen clearly from Figure 5(a1) that inside the microspheres, there were a lot of small scattered particles, showing that mass transfer between the droplets and the ambient solution was taking place, but the second layer had not formed yet at that moment. The double emulsion droplets were cured to form core-shell microspheres, as shown in Figure 5(a2). Additionally, we also prepared microspheres with three layers, as shown in Figure 5(a3). Then, we rinsed the microspheres using DI water to remove the PVA around the microspheres and observed them in a scanning electron microscope. As shown in Figure 5(b1), the microspheres had good monodispersity and sphericity. Figure 5(b2,b3) are magnified SEM images, and it could be found that the surface of the microspheres was solid, smooth, and dense. Figure 5c shows the SEM images of a cross-section of microspheres with different layers, which clearly shows the monolayer structure (Figure 5(c1)), core-shell structure (Figure 5(c2)), and triple-layer structure (Figure 5(c3)). Here, the third layer is marked with a red line in the image.

### 3.3. The Effect of Droplet Size on Phase Separation Process

**Figure 6:**
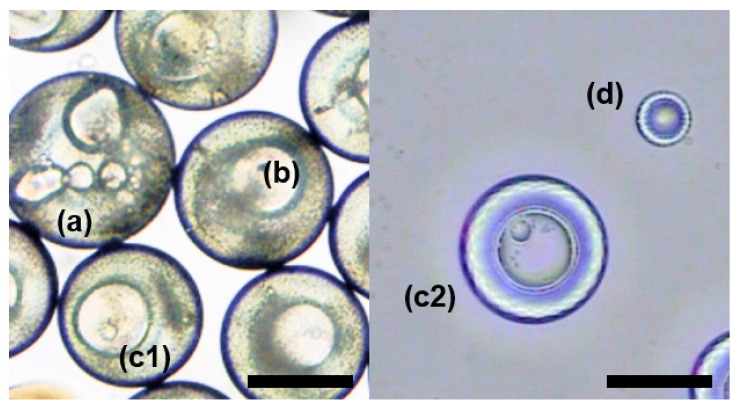
Droplets of different sizes. Droplets were denoted as (**a**–**c**) and (**d**) from big to small, in which (**c1**) was the same size as (**c2**). (a) was a microsphere with many small cores; (**b**) was a microsphere with two layers; (**c1**,**c2**) were microspheres with three layers; (**d**) was a single emulsion. The scale bar is 200 µm.

In the process of droplet preparation, we found that the droplet size had an influence on the phase separation process. Phase separation proceeded more slowly when the microspheres were slightly larger. Hence, we prepared droplets with different sizes and observed their structure after the same amount of time, as shown in Figure 6. Droplets were denoted as a, b, c, and d from big to small, in which (c1) was the same size as (c2). The droplets with different sizes would be in different stages of phase separation after the same time and showed different structures. As shown in Figure 6, the droplet (a) had just started to change and was in the core formation stage, droplet (b) was double emulsion, droplet (c1) and (c2) formed a triple-layered structure, and droplet (d) had finished phase separation and presented the final single emulsion droplet.

**Figure 7:**
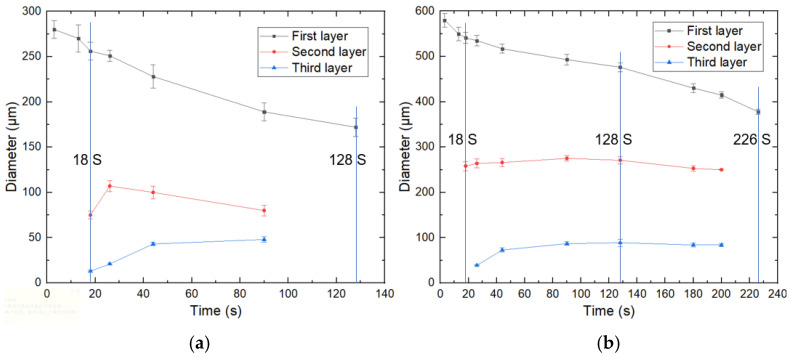
Diameter variation of each layer of droplets with different initial sizes: (**a**) 280 μm; (**b**) 580 μm.

Droplets with two initial sizes (280 μm and 580 μm) were studied specifically, and the diameters of each layer of droplets at different times were recorded, as shown in Figure 7a,b. It could be seen that the size variation trend of the two microspheres was similar. The diameter of the first layer gradually decreased, the diameter of the second layer increased and then decreased, and the diameter of the third layer gradually increased. The difference was that the small-size microsphere had formed the third layer at 18 s (S), while the large-size microsphere still had two layers. The small-size microspheres had completed phase separation and became single emulsion droplets at 128s. In contrast, the large-size microsphere still had three layers at this time and did not become single emulsion droplets until 226 s. In the previous work performed by Liang et al. [40], the mass transfer velocity of an emulsion droplet was determined by its surface area, which was proportionate to the diameter of the pristine single emulsion droplet (*d_0_*), and the mass transfer velocity per unit volume of the droplet should be proportionate to 1/*d_0_*. Therefore, this tendency could be understood easily.

### 3.4. Drug Controlled Release of Microspheres

**Figure 8:**
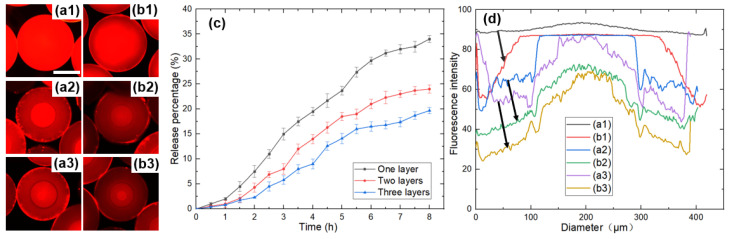
(**a**) Fluorescence images of microspheres with different number of layers: (**a1**) One layer, (**a2**) Two layers, (**a3**) Three layers. (**b**) Fluorescence images of microspheres with different number of layers after drug release: (**b1**) One layer, (**b2**) Two layers, (**b3**) Three layers. The scale bar is 200 µm. (**c**) Drug release profiles of microspheres. (**d**) Corresponding fluorescence intensity along the diameter of the microspheres before and after drug release. The letters correspond to the microspheres in (**a**,**b**).

DOX is a hydrophilic fluorescence indicator with red fluorescence, which could be automatically assembled into the hydrophilic areas during the multistep phase separation. It was also easy to monitor the behavior of the drug in the microspheres by fluorescent microscopy and visualize its distribution in microspheres. The fluorescence images of microspheres with one layer, two layers, and three layers are shown in Figure 8(a1–a3), respectively.

In order to simulate the process of microspheres moving from the stomach into the intestine, the DOX-loaded microspheres were dispersed into 4 mL of SGF for 2 h, and after 2 h, the microspheres were transferred to 4 mL SIF for 6 h at room temperature. The fluorescence images of microspheres with one layer, two layers, and three layers after drug release are shown in Figure 8(b1–b3), respectively. The drug concentrations were determined at regular intervals, and the profiles of DOX released from microspheres with different layers are shown in Figure 8c. The corresponding fluorescence intensity along the diameter of the microspheres before and after drug release is shown in Figure 8d, and it can be seen that the fluorescence intensity reduced, indicating that the drug released from the microspheres into the ambient solution. The figure shows that the DOX released from microspheres over time, and it can be seen that the release behavior of microspheres with different layers was variable, among which the microspheres with one layer showed the fastest release, the microspheres with two layers released second, and the microspheres with three layers released the slowest.

As mentioned in Section 3.1, the mass transfer between PEGDA 700 and the external solution resulted in an increase in the number of layers of microspheres, as well as the proportion of PEGDA 250 in the microspheres. The lower PEGDA molecular weight resulted in a denser and more tightly crosslinked system [46,48,49], inhibiting drug release and leaving the DOX molecule trapped within the microspheres. In contrast, higher molecular weight PEGDA systems resulted in faster drug release. Figure 8(b1) shows that the drug was released from the edge of the microsphere. When the microsphere became two layers, the drug was mainly distributed in the inner layer and therefore needed to diffuse through the dense outer layer to the external environment. When the microspheres became three layers, the innermost drug was trapped by the dense structure. Therefore, it was not easy to release the drug in the innermost layer, showing slightly more brightness than the second layer after release.

### 3.5. Preparation of “Cup-Shaped” Microparticles

**Figure 9:**
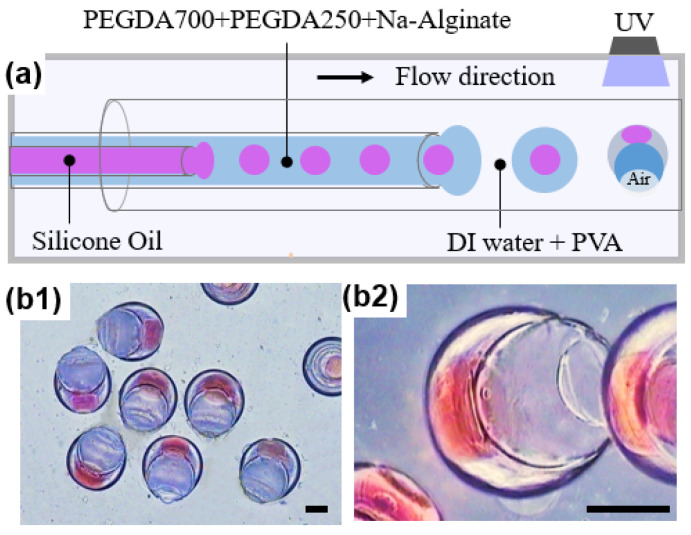
(**a**) Schematics of the microfluidic device used for preparing “cup-shaped” microparticles capable of encapsulating oil cores. The initial double emulsion droplets gradually formed an open hollow structure due to phase separation. (**b**) Optical microscopic images of “cup-shaped” microparticles. (**b1**) Optical microscopic image showing good monodispersity and uniform size and shape of microparticles; (**b2**) Magnified image showing the open hollow structure of the microparticle with an oil core. Silicone oil was dyed red. The scale bar is 200 µm.

In addition to loading hydrophilic drugs during phase separation, we also used a microfluidic device with three capillary tubes to prepare microparticles capable of encapsulating oil cores. All of the tubes were set in good alignment to form a coaxial geometry, as shown in Figure 9a. The inner phase consisted of silicone oil dyed red, the middle phase was composed of 50 *v*/*v*% PEGDA 250, 40 *v*/*v*% PEGDA 700, and 10 *v*/*v*% Na-Alginate aqueous solution, and the outermost phase was 5 wt% PVA aqueous solution. It was found that the oil core can be wrapped to form double emulsion droplets at the beginning. However, with the mass transfer between PEGDA 700 in the intermediate and outermost solutions, PEGDA 700 gradually converges and nucleates and was expelled by the PEGDA 250 shell because of interfacial tension and the presence of the oil core [50]. Then, the particles gradually shrank into a shape with an open hollow structure, which was also known as the “cup” shape [51], as shown in Figure 9b. The large cavity and appropriate openings enable microparticles to have potential applications in the encapsulation and release of hydrophobic drugs, as well as enhancing cancer therapies [51].

## 4. Conclusions

In this work, we developed a microfluidic device with a co-flow structure to fabricate microspheres with different layers based on mass transfer-induced phase separation. The structure of solid microspheres could be controlled by UV treatment at different times during the liquid–liquid phase separation process. From the release curves, it could be seen that the microspheres showed a controllable release of DOX in SGF and SIF. In the reported data, the microspheres with more layers had lower release due to more PEGDA 250 in them, indicating that this process had the capability to control the drug release by varying the number of layers in the microspheres. These features of the microspheres showed their great potential applications in drug delivery and other fields.

## Data Availability

The data presented in this study are available on request from the corresponding author J.M.
